# Socio-economic determinants of child mortality in Pakistan and the moderating role of household’s wealth index

**DOI:** 10.1186/s12887-021-03076-2

**Published:** 2022-01-03

**Authors:** Muhammad Farhan Asif, Zahid Pervaiz, Jawad Rahim Afridi, Rida Safdar, Ghulam Abid, Zohra S. Lassi

**Affiliations:** 1 Department of Economics, National College of Business Administration and Economics, Lahore, Pakistan; 2 Department of Economics, Sarhad University of Information Technology, Peshawar, Pakistan; 3 Shaikh Khalifa Bin Zayed Al-Nahyan Medical and Dental College, Lahore, Pakistan; 4 Department of Business Studies, Kinnaird College for Women, Lahore, Pakistan; 5 Robinson Research Institute, The University of Adelaide, Adelaide, Australia

**Keywords:** Child mortality, Pakistan Demographic and Health Survey, Pakistan

## Abstract

**Background:**

Child mortality is an important social indicator that describes the health conditions of a country as well as determines the country’s overall socio-economic development. The Government of Pakistan has been struggling to reduce child mortality (67.2 per thousand live births in 2019). Pakistan could not achieve the target set for Millennium Development Goals to reduce child mortality and still working to meet the target set by the Sustainable Development Goals. This study has investigated the socio-economic determinants of child mortality in Pakistan by using household-level data. Socio-economic characteristics related to women (mothers) and households have been considered as possible determinants of child mortality. The moderating role of a household’s wealth index on the association between woman’s education and child mortality has also been investigated.

**Methods:**

The comprehensive dataset of the Pakistan Demographic and Health Survey 2017–18 has been used to explore the determinants of child mortality by using multivariable logistic regression. The interaction term of women’s education and household wealth index has been used to investigate the moderating role of the household’s wealth index.

**Results:**

The results indicate that the likelihood of child mortality decreases with an increase in women’s education, their empowerment, their husband’s education, the wealth status of their households, access to clean drinking water, access to toilet facilities, and exposure to mass media. Whereas, an increase in unmet need for family planning increases the likelihood of child mortality. The study also identified the moderating role of a household’s wealth index on the association between woman’s education and child mortality.

**Conclusions:**

Household wealth status moderates the association between women’s education and child mortality. The absolute slope of the curve showing the association of women’s education and child mortality is higher (more negative) for richer households than poorer households. It implies that a household’s wealth status strengthens the relationship between women’s education and child mortality. With the increase in the household’s wealth status, the effect of a mother’s education on child mortality becomes more pronounced.

## Background

Child mortality is one of the most commonly used indicators of the socio-economic development of a population. It can be defined as “the likelihood for a child, born alive, to die before the age of five years” [1]. The health status of the population determines the socio-economic development of a nation [2]. The right to health is the fundamental part of the Human Rights Declaration of 1948 [3] whereby it is a moral duty of a country’s policymakers to work for the improvement of the health of their people. For future human capital, children are a precious asset and they deserve a better and healthy life. This can only be possible if the children survive in the early years of their life [4]. Around 5.2 million children, in 2019, died before reaching the age of five years globally. The issue is even more severe in developing countries [5]. The rate of child mortality is 18 times higher in low and middle-income countries (LMICs) as compared to the high-income countries (HICs) [6]. According to Lozano et al. (2011) [7], 50% of the total worldwide incidents of child mortality occur in five countries i.e. India (22%), Nigeria (13%), Pakistan (6%), the Democratic Republic of the Congo (6%), and China (4%).

Pakistan is one among the developing countries where overall health conditions are poor as it ranks at 154^th^ position among 195 countries in terms of accessibility and quality of healthcare [7]. The country ranked at 36^th^ position out of 228 countries for child mortality in 2018, which is an indication of the worse situation of child health. Although, Pakistan has made some progress in a reduction in child mortality which has decreased from 141 per thousand live births in 1990 to 67.2 per thousand live births in 2019 [5] yet it is still higher than developed and even many developing countries. Thus, the issue of child mortality is required to be analyzed more deeply in the context of Pakistan.

Historically, the decline in infant and child mortality around the world can be attributed to different factors such as improvements in medical technology, dissemination of health knowledge, and improvements in living standards [8]. Child mortality is expected to be influenced by biological factors (age of mother, birth interval, birth order (male/female baby), and weight of the child at the time of birth) family structure (joint/nuclear) as well as socio-economic (wealth status of household, education and employment status of parents, etc.) [9–15], and cultural factors (food habits, set of values and ideologies of a particular community) [7, 8, 14–18].

Researches have investigated various demographic, socio-economic (1, 17), and ecological or environmental factors [19] which have significant effects on child and infant mortality [17, 20–26]. The empirical evidence of the relationship of different socio-economic factors with child and infant mortality in the context of Pakistan is also available [22, 27–30]. Women’s education is found to be an important predictor of child mortality among many other socio-economic factors. Child mortality is negatively affected by women’s education [17, 31]. This is because a well-educated mother can be expected to take care of her child and herself during and after the pregnancy in a better way as compared to an uneducated or less educated mother due to better knowledge and awareness about health and prevention of diseases. The role of socioeconomic status (SES) of women and their households can also be an important determinant of child mortality [32]. This study has investigated the socio-economic determinants of child mortality in Pakistan by using the household level data of Pakistan Demographic and Health Survey (PDHS) 2017–18. Socio-economic characteristics related to women (mothers) and households have been considered as possible determinants of child mortality. The study has also endeavored to examine the moderating role of a household’s wealth index on the association between women’s education and child mortality. The multi-dimension factor associated with child mortality might be done.

## Methods

### Data source

**Fig. 1:**
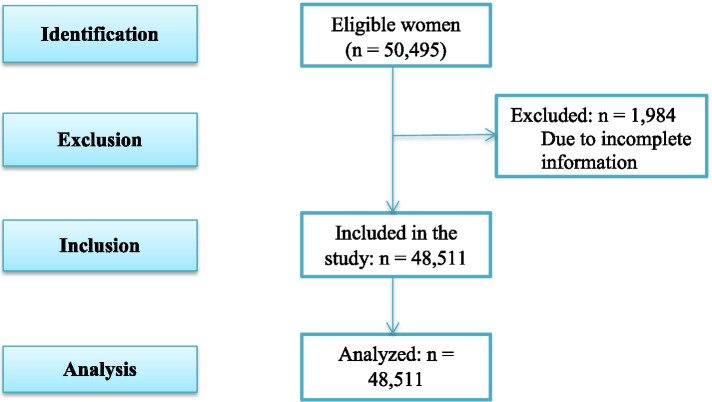
Strobe flow diagram

The data for the current study were taken from PDHS 2017–18. A two-stage sampling technique was used by PDHS to collect the data. In the first stage, a systematic sampling technique was used in which 580 sampling units were selected across the country. Out of these 580 sampling units, 295 were from rural areas and 285 were from urban areas. At the second stage, an equal probability systematic method was adopted in which a fixed number, i.e., 28 households were randomly chosen from all the clusters. Thus, the sample size consisted of 16,240 households out of which 8260 were residing in rural areas whereas 7980 were from urban areas. A total of 50,495 married women were interviewed during the time span of the year 2017–2018 [33]. PDHS data set consisted of four different files i.e. household characteristics, women, men, and child characteristics. We examined the complete data set to understand the measures, actual items, or questions in the survey and the way the data were collected and/or documented. Then, we extracted the data related to all the study indicators from the household characteristics data set. After removing the missing data, we had 48,511 observations for our analysis (Fig. 1).

### Measurement

We have considered the different socio-economic factors as determinants of child mortality. The functional form of the model used in our study is given below.

$$\mathrm{CM}=\mathrm{f}(\mathrm{HWI},\mathrm{ WEDU},\mathrm{ HEDU},\mathrm{ WEMP},\mathrm{ UMNFP},\mathrm{ EMM},\mathrm{ APW},\mathrm{ ATF},\mathrm{ WEDU}*\mathrm{HWI}).$$

CM = Child Mortality used as predicted variable in our research. It has been used as a binary variable. Coded as 1 if there has been the prevalence of child mortality in the household and 0 otherwise.

HWI = Household wealth index has been constructed to assess the wealth status of the households. A wealth score has been assigned to each household which has been calculated by using Principal Component Analysis and taking into account the household's ownership of selected assets, such as television, refrigerator, motorcycle, car, tractor, and bicycles; water and sanitation facilities available to households, such as type of toilet and source of drinking water; materials used for housing construction such as wall material, flooring material, and roof material; type of fuel used for cooking, such as electricity, natural gas, kerosene oil, coal, agricultural crop or animal dung, etc. Based on the score of the wealth index, households have been placed in 5 different quintiles. Households belonging to the fourth and fifth quintiles have been considered as wealthy households and coded as 1 whereas households belonging to the first three quintiles have been coded as 0.

WEDU = Women’s education has been reported in PDHS as four different categories i.e. no education, primary, secondary and higher education. This information has been used as a binary variable divided into two categories i.e. less than secondary coded as 0 and at least secondary education coded as 1.

HEDU = Husband’s education has been categorized into four different categories i.e. no education, primary, secondary and higher education in PDHS. The information about the husband’s education has been used to construct a binary variable for the husband’s education; coded as 1 in the case of at least a secondary school education and 0 otherwise.

WEMP = Women empowerment used as a binary variable. Women’s involvement in decision-making regarding household consumption has been used as a proxy for women's empowerment. This variable has been used as a binary variable. The woman is considered empowered if she has involvement in the decision regarding household consumption (she decides consumption alone or jointly with her husband or with any other family member). She is considered not empowered if she does not have any involvement in decision-making regarding household consumption.

UMNFP = Unmet need for family planning classified into two categories as women having UMNFP and women not having UMNFP. Women are considered to have UMNFP if, despite their intention to use family planning methods, they do not do so due to some reason.

EMM = Exposure of mass media, the existence of television (TV) at household has been used as a proxy for this variable. It is classified into two categories; coded as 1if household owns TV and 0 otherwise.

APW = Access to protected/safe water is divided into two categories i.e. coded as 1 if the household has access to protected/safe water and coded as 0 if the household does not have access to protected/safe water.

ATF = Access to toilet facility is divided into two categories; coded as 1 if the household has access to a toilet facility and 0 otherwise.

WEDU*HWI = Interaction term of women’s education and household wealth index. The interaction term has been used to investigate the moderating effect of the household wealth index on the association of women’s education and child mortality.

### Statistical analysis

Multivariable logistic regression has been used to investigate the socio-economic determinants of child mortality. Step-wise regression has been applied to select the variables of our model. The stepwise regression has been run on thirteen independent variables (household wealth index, woman’s education, husband’s education, woman’s empowerment, unmet need for family planning, exposure to mass media, access to safe drinking water, access to toilet facility, place of residence, household headship, women’s employment, husband’s occupation status and the number of living children) which could be possible determinants of child mortality. Out of thirteen variables, eight variables (household wealth index, woman’s education, husband’s education, woman’s empowerment, unmet need for family planning, exposure to mass media, access to safe drinking water, and access to toilet facility) have been chosen for further analysis. For this purpose, we used SPSS software version 20.

To investigate the moderating effects of the household wealth index, an interaction term of women’s education and household wealth index has been used. The interaction term is used when the impact of an independent variable on a dependent variable can be explained with the help of a third variable which is termed as moderating variable. The moderating variable can strengthen or weaken the existing impact of the independent variable on the dependent variable. As we are interested to investigate the moderating role of the household wealth index, therefore, we have used interaction terms of women’s education and household wealth index. SPSS software version 20 has been used for the analysis. We have also used the bootstrapping technique i.e. Process by Hayes (as a supplementary analysis) to investigate the moderating role of wealth index on the relationship between women’s education and child mortality [34]. It is a well-known resampling technique that evaluates the factors of the model and the standard errors from the sample. For this purpose, we used PROCESS Macro software. [35–37].

## Results

Descriptive statistics of variables of the study are presented in Table 1. 7.6% of the total respondent women had experienced the incidence of child mortality. More than half of the total women were not empowered (55%) and had no exposure to media (57.1%). About three fourth of the total women (74.2%) and half of the women’s husbands (47.3%) had an education of less than secondary school. 66% of the households were poor, three fourth of them had no access to safe drinking water (75.1%) and more than three quarters had access to a toilet facility (83.9%). One-fifth of the women had an unmet need for family planning (20.9%).

**Table 1:** Socio-demographic characteristics of included participants (*n* = 48,511)

Descriptive	Frequency	Percent (%)
Child’s living status		
Alive	44,838	92.4
Not alive	3,673	7.6
Household wealth index		
Poorer	32,138	66.2
Richer	16,373	33.8
Woman’s education		
Less than secondary	35,968	74.2
At least secondary	12,543	25.8
Husband’s education		
Less than secondary	22,932	47.3
At least secondary	25,579	52.7
Woman’s empowerment		
Less empowered	26,703	55.0
More empowered	21,808	45.0
Unmet need for family planning		
No	38,377	79.1
Yes	10,134	20.9
Exposure to mass media		
No exposure	27,685	57.1
Exposure	20,826	42.9
Access to safe drinking water		
No	36,425	75.1
Yes	12,086	24.9
Access to toilet facility		
No access	7,794	16.1
Access	40,717	83.9

The outcomes of logistic regression (Table 2) illustrate that women's education (OR = 0.788), women empowerment (OR = 0.948), husband’s education (OR = 0.838), household wealth index (OR = 0.988), exposure to mass media (OR = 0.953), access to toilet facilities (OR = 0.815), and access to protected/safe drinking water (OR = 0.908) are negatively and significantly related to child mortality. The likelihood of child mortality is lower in women who have attained at least secondary school education, are empowered, have exposure to mass media, and whose husbands are educated. Such likelihood is also lower in the case of wealthier households with access to safe/protected water and toilet facility. The met need for family planning (OR = 0.901) is negatively related to child mortality. The likelihood of child mortality is lower among women who do not have UMNFP. The odds ratio (OR = 0.786) shows that the association between women's education and child mortality is more negative for richer than poorer households.

**Table 2:** Predictors of child mortality using multivariable logistic regression

Variables	Odds Ratio (OR)	Class Interval (C.I)		Sig
		**Lower C.I**	**Upper C.I**	
Household wealth index				
Poorer	Reference			
Richer	0.988	0.779	1.084	0.026
Woman's education				
Less than secondary	Reference			
At least secondary	0.788	0.717	0.959	0.038
Husband’s education				
Less than secondary	Reference			
At least secondary	0.838	0.769	1.186	0.000
Woman empowerment				
Less empowered	Reference			
More empowered	0.948	0.669	1.371	0.028
Unmet need for family planning				
Yes	Reference			
No	0.901	0.822	1.108	0.015
Exposure to mass media				
No	Reference			
Yes	0.953	0.869	.1.018	0.024
Access to safe drinking water				
No	Reference			
Yes	0.908	0.789	0.994	0.000
Access to toilet facility				
No	Reference			
Yes	0.815	0.766	0.931	0.000
Woman’s education * wealth index	0.786	0.493	0.927	0.016

**Fig. 2:**
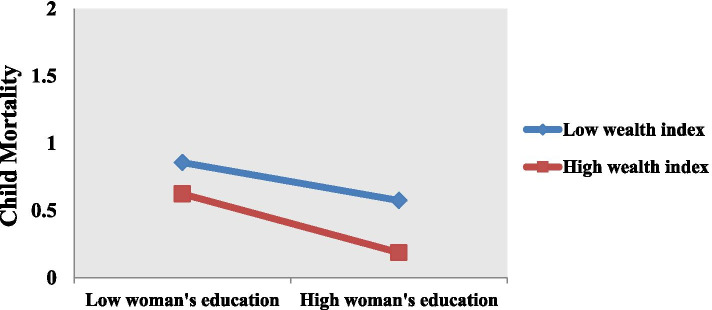
Graphical representation of moderation analysis

The supplementary analysis for moderating effect using Process Hayes has also shown that the interaction effect of women education and household wealth index (β = -0.187, *p* < 0.01) is significantly reducing for child mortality (Table 3). Slope analysis is executed to further illustrate the moderating effects of the household wealth index on the relationship between women’s education and child mortality (Fig. 2). The absolute slope of the curve showing the association of women’s education and child mortality is steeper downward (more negative) for richer households than poorer households. It implies that a household’s wealth status strengthens the relationship between women’s education and child mortality. With the increase in the household’s wealth status, the effect of a mother’s education on child mortality becomes more pronounced.

**Table 3:** Results of Moderation Using Process Hayes

Variables	β	Class Interval (C.I)		Sig
		**Lower C.I**	**Upper C.I**	
Constant	1.163	0.173	1.553	0.000
Woman’s education	-0.146	-0.155	-0.105	0.008
Wealth index	-0.102	-0.114	-0.059	0.000
Woman’s education * wealth index	-0.187	-0.364	-0.039	0.042

## Discussions

The analysis shows that women’s education, women’s empowerment, and exposure to mass media are important factors to bring a reduction in child mortality. Women’s education can have several benefits for themselves, their families, and society. Educated women are more likely to participate in the labor force actively, have lower fertility, and have children who are well-nourished and healthier. They are expected to have better knowledge and awareness about hygienic practices and preventive health care. They can also be in a better position to take care of themselves and their children during and after pregnancy [17, 24, 31, 38]. It is quite plausible to expect that women’s education would play a crucial role to decrease child mortality. Thus, the economic benefits of addressing and reducing barriers to women’s education and engagement in the workforce can be substantial [39–43].

Educated women are generally more empowered and expected to have more involvement in decision-making at the household level. Women who are empowered, acquire greater bargaining power, and have more decision-making autonomy are likely to have lower fertility rates [44], and higher use of contraceptives. Female autonomy in decision-making decreases unwanted births up to 57% [45]. Women’s control over household consumption leads to more spending on children’s education, health, and nutrition. It has also been noted that if both husband and wife contribute to household income then the larger share of women’s income is spent on children’s health [44] and nutrition [46, 47]. Maitra (2004) [48] reports that more female autonomy results in a significant reduction of child mortality because of the greater prenatal care and more possibility of delivery in the hospital.

Women’s exposure to mass media is important to reduce child mortality as it helps them to have necessary and useful information regarding child vaccination and proper health facilities that must be provided to the children. Because of this awareness, mothers can take good care of their children which reduces the risk of child mortality [27, 49]. Husband’s education is inversely associated with child mortality. An educated individual is likely to have a better income which plays a key role in the financial strength of the family and the provision of necessary health facilities for the family [50, 51]. An educated man is also more likely to be well aware, knowledgeable, and conscious about the importance of child health. The household wealth index is negatively associated with child mortality. Wealthier households have better living standards and better availability of health facilities. This can help to reduce the likelihood of child mortality among wealthier households. On the other hand, the households with lower wealth status are less likely to have availability of health facilities and access to basic facilities of sanitation, hygienic toilets, and clean drinking water [22, 52].

Access to toilet facilities reduces the likelihood of child mortality by reducing the prevalence of diarrhea which is one of the main causes of child deaths [53]. The diarrheal disease was the reason for every tenth child’s death and caused 0.53 million deaths per year worldwide in 2017 [54]. Water, sanitation, and hygiene (WASH) involvements are the best methods to avert diarrheal diseases [55] which can help to reduce child mortality [50].

UMNFP is positively associated with child mortality. The prevalence of UMNFP in Pakistan can be attributed to several socio-economic and cultural factors. Fear of side effects and socio-cultural norms are among some important factors restricting women’s access and use of contraceptives. Effective public policy interventions and social mobilization through the involvement of religious and community leaders can be helpful to reduce UMNFP [56, 57]. The moderating effect of a household’s wealth index on the association of women’s education with child mortality is significant. Household wealth status strengthens the relationship between women’s education and child mortality. With the increase in the household’s wealth status, the effect of a mother’s education on child mortality becomes more pronounced. It implies that a household’s wealth status remains a crucial factor to reduce child mortality. It seems quite plausible as a household’s wealth status determines their access to facilities of health. Ensuring equitable access to health care services through effective public policy can be helpful to reduce the gaps in health outcomes among different sections and classes.

### Limitations of the study

The study has investigated the socio-economic determinants of child mortality in the context of Pakistan. It has also analyzed the moderating role of household wealth status in the relationship between women’s education and child mortality. However, child mortality can be affected by a number of other factors which need to be investigated. Women’s employment status and their place of residence (rural/urban) can also play a moderating role in the relationship between women’s education and child mortality.

## Conclusions

Pakistan is a developing country where poverty is one of the important and most pressing issues. The prevalence of poverty coupled with poor facilities of sanitation and health, lower levels of education; particularly lower levels of female education, and poor socio-economic conditions of people have led to a higher prevalence of child mortality. Inadequate food, malnutrition, unhygienic living conditions, and lack of access to health care facilities are expected to be inevitable outcomes of poverty. Effective public policy aiming to uplift the socio-economic status of poor people and ensure the availability of health and sanitation facilities can be an important option to cope with the problem of child mortality in the country.

## Data Availability

We have used the secondary data of PDHS 2017–18. Available at: https://www.nips.org.pk/study_detail.php?detail=MTgw

## Abbreviations

LMICs: Low and middle-income countries
HICs: High-income countries
SES: Socioeconomic status
UMNFP: Unmet need for family planning
PDHS: Pakistan Demographic and Health Survey
WASH: Water, sanitation, and hygiene
